# Retraction: Effects of UCP4 on the proliferation and apoptosis of chondrocytes: Its possible involvement and regulation in osteoarthritis

**DOI:** 10.1371/journal.pone.0324041

**Published:** 2025-05-07

**Authors:** The PLOS ONE Editors

After this article [1] was published, concerns were raised about Figs 1, 4, 5, and compliance with the PLOS Animal Research policy.

Specifically:

- The UCP4 panel in Fig 1B appears similar to the Bcl-2 panel in Fig 4C of [2] and the MMP2 panel of Fig 6B in [3].
- The GAPDH panel in Fig 4B appears similar to the GAPDH panel in Fig 2B of [4] when aspect ratio is altered.
- The GAPDH panel in Fig 5C appears similar to the GAPDH panel in Fig 9e of [5].
- In Fig 5E, there appear to be vertical discontinuities between lanes 1–2 in the Cleaved Caspase-3 panel, lanes 1–2 in the Bax panel and between lanes 2–3 of the PCNA panel, when color levels are adjusted to visualize the background.

The corresponding author acknowledged that there are inconsistencies in the data and that they have been unable to verify or replicate the data through subsequent experimentation. No underlying data were provided and as such the concerns remain unresolved.

The corresponding author also stated that they identified concerns related to the adherence to animal welfare standards in [1], specifically, that the use of 10% chloral hydrate for anesthesia and the euthanasia methods employed may not fully align with internationally accepted standards, and they requested retraction of the article.

In light of the above concerns, and in accordance with the request of the corresponding author, the *PLOS ONE* Editors retract this article.

PT agreed with the retraction. ZH, JL, SD, GC, YQ, LH, and LX either did not respond directly or could not be reached.

The GAPDH panel in Fig 5C is similar to material from [5], published in 2015 by Springer Nature, which is not offered under a CC-BY license and is therefore excluded from this article’s [1] license.
